# Crystal structure and supra­molecular features of a bis-urea-functionalized pillar[5]arene

**DOI:** 10.1107/S2056989023009003

**Published:** 2023-10-19

**Authors:** Mickey Vinodh, Fatemeh H. Alipour, Talal F. Al-Azemi

**Affiliations:** a Department of Chemistry, Kuwait University, PO Box 5969, Safat 13060, Kuwait; Universidad Nacional Autónoma de México, México

**Keywords:** pillararene, urea substitution, receptor, crystal structure, dimer

## Abstract

The crystal structure of a bis-urea derivative based on A1/A2-functionalized pillar[5]arene (**DUP**) that encapsulates dimethyl formamide (**DMF**) inside the macrocyclic cavity is reported. The crystal structure of **DUP·DMF**, C_63_H_70_N_4_O_12_·C_3_H_7_NO, reveals that out of two urea functionalized spacers, one arm is oriented above the macrocyclic cavity with strong hydrogen-bonding inter­actions between the urea H atoms and **DMF** guest, whereas, the other arm is positioned away from the macrocycle, leading to inter­molecular hydrogen-bonding inter­actions between the urea H atoms of two adjacent pillar[5]arene macrocycles, resulting in the formation of a supra­molecular dimer.

## Abstract 2

The crystal structure and supra­molecular features are reported of a dimeric bis-urea-functionalized pillar[5]arene macrocycle, which functions as a receptor system to the DMF guest mol­ecule.

## 1. Chemical context

The design of mol­ecular receptors based on pillararenes is an active research area (Ogoshi & Yamagishi, 2013 ▸; Ogoshi *et al.*, 2016 ▸; Fang *et al.*, 2020 ▸). In particular, pillararene receptors bearing multiple urea-based substituents that possess polarized N—H groups are important derivatives in the field of mol­ecular recognition and sensing because of their excellent guest–host inter­actions (Duan *et al.*, 2012 ▸; Ni *et al.*, 2014 ▸; Feng *et al.*, 2017 ▸). The presence of strong hydrogen-bonding inter­action sites in the macrocyclic rim provided by the presence of N—H groups is the prime factor for determining the efficiency of such host–guest inter­actions, and consequently, the extent of their mol­ecular recognition ability. As a result, the number and relative position of the N—H groups with respect to the pillararene macrocycle is very crucial in such mol­ecular receptors. Recently, we have reported the synthesis of urea-functionalized anionic receptors based on di- and tetra-functionalized pillar[5]arenes (Vinodh *et al.*, 2023 ▸). The influence of the receptor structure on the selectivity and binding ability toward different halides was investigated by ^1^H NMR titrations, diffusion-order spectroscopy (DOSY) and isothermal titration calorimetry (ITC) experiments. It was observed that the non-covalent inter­actions between the receptors and the guest anions are affected by both the number of the urea substituents and their relative positions on the pillar[5]arene frame. In addition, the supra­molecular self-assembly mediated by hydrogen-bonding inter­actions of urea-functionalized substituents on the pillararene frame in solution was also detected. Therefore, a detailed crystal-structure determination of bis-urea-functionalized pillararenes is very important for obtaining more insight into their mol­ecular recognition characteristics. In the present communication we report the single-crystal X-ray structure of an inclusion complex of A1/A2-bis-urea functionalized pillar[5]arene (**DUP**) with a **DMF** mol­ecule. The structural details, host–guest inter­actions and other supra­molecular features of this macrocyclic system (**DUP·DMF**) were investigated and are discussed in detail. 
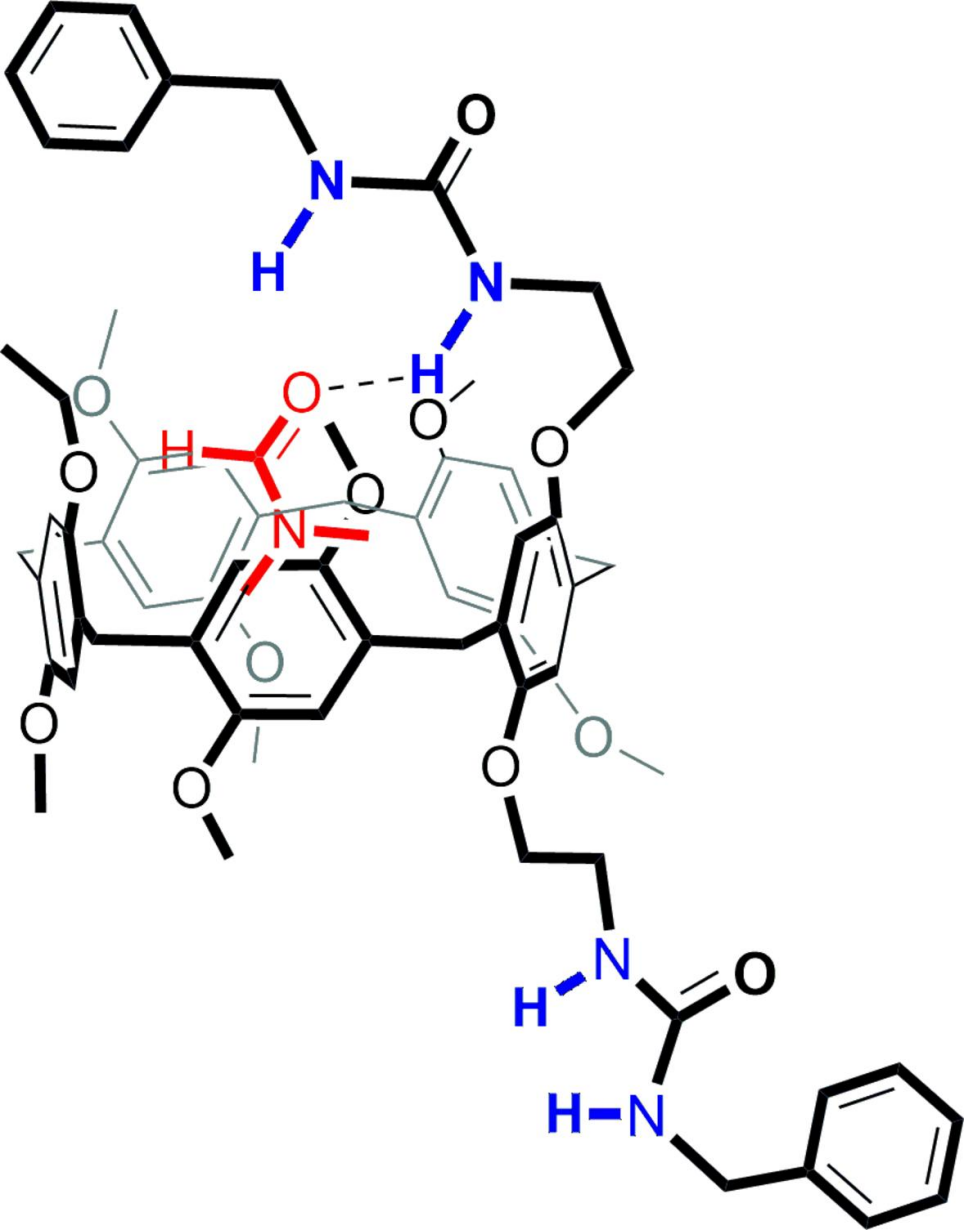


## 2. Structural commentary

**Figure 1:**
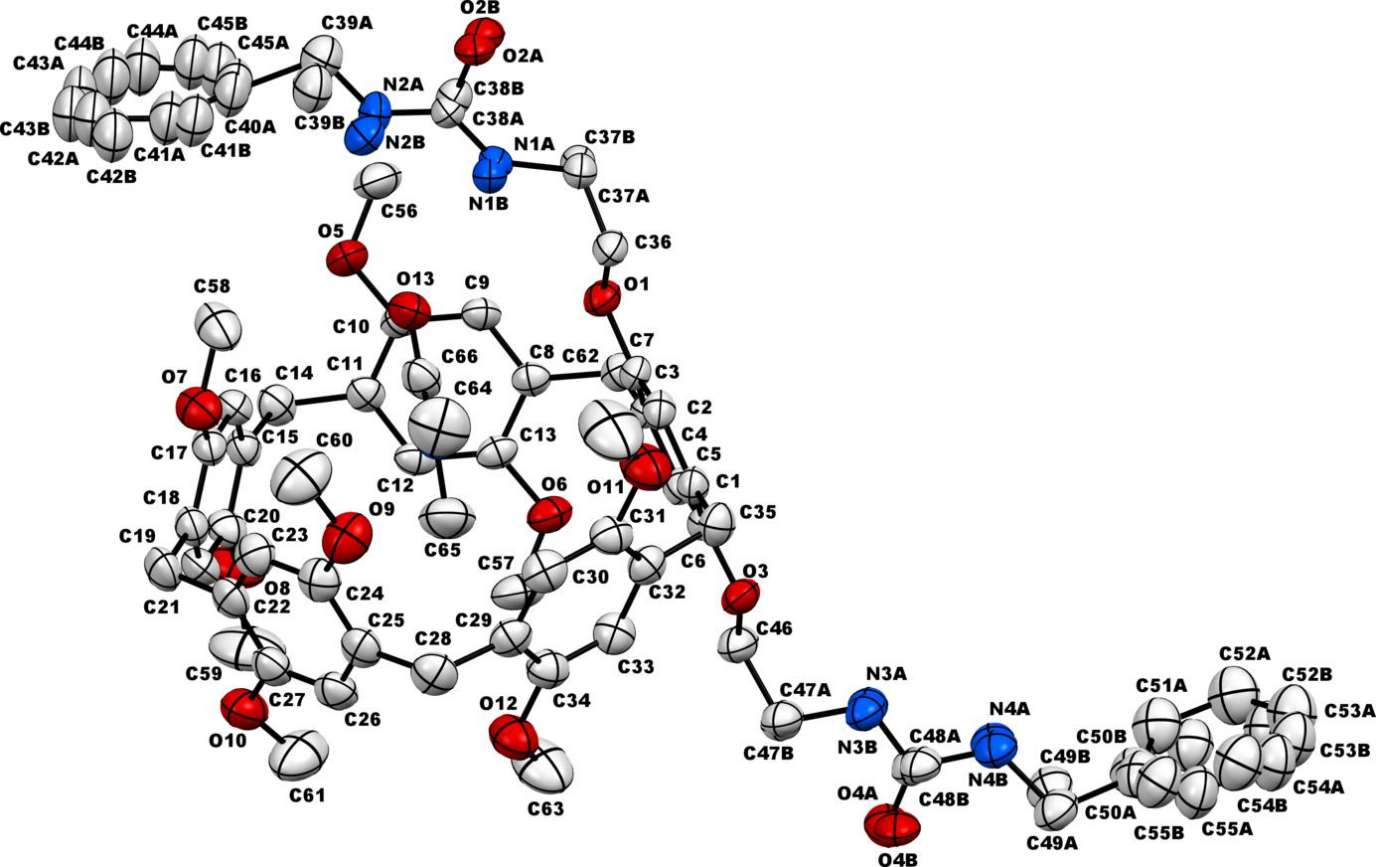
Crystal structure of **DUB·DMF** with displacement ellipsoids at the 30% probability Hydrogen atoms are omitted for clarity.

**Figure 2:**
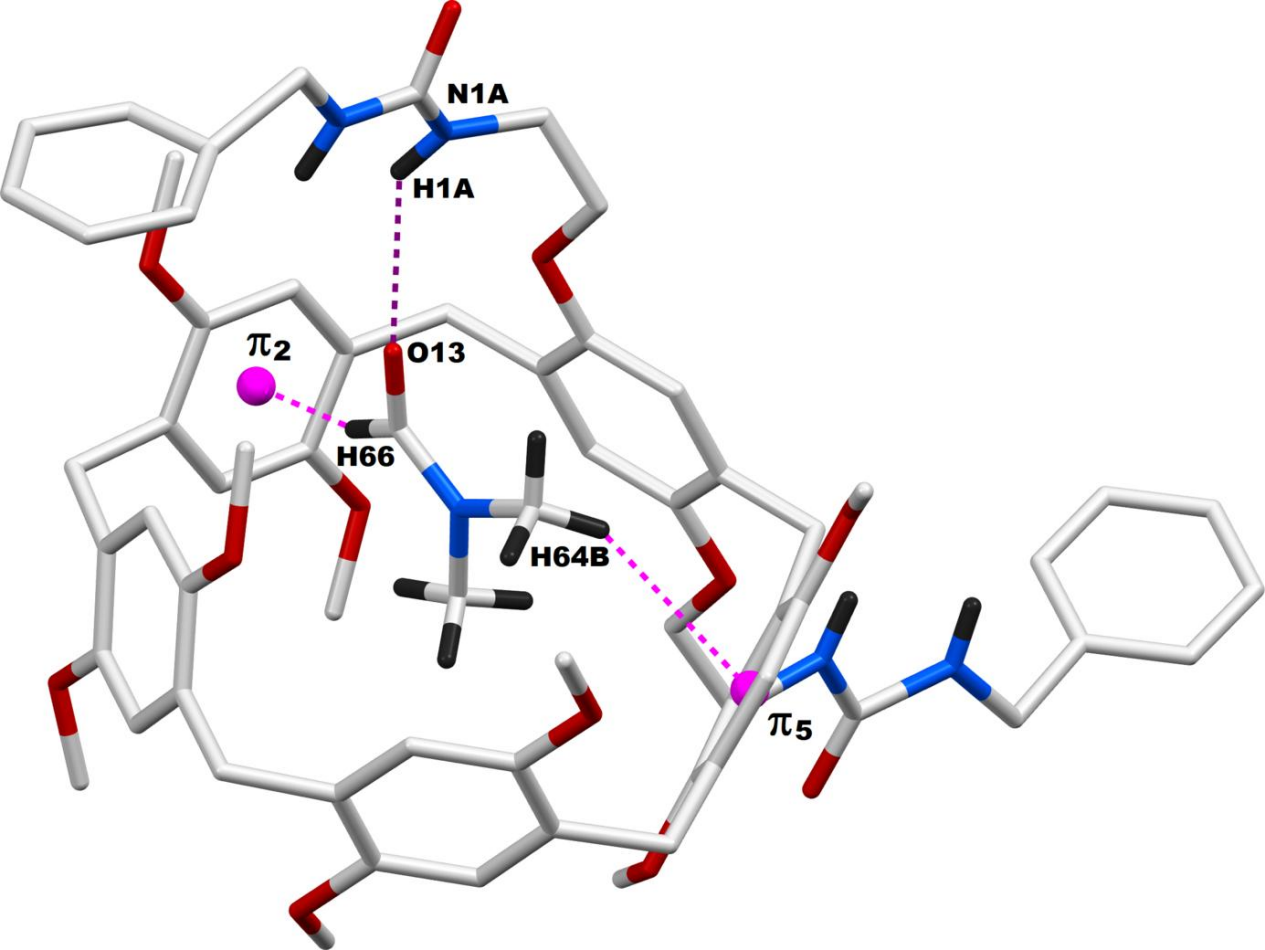
Inter­molecular inter­actions between the pillar[5]arene host and the DMF guest; π2, and π5 are the centroids of the phenyl rings C8–C13 and C29–C34, respectively. Hydrogen atoms except those on urea moieties and the DMF mol­ecule are omitted for clarity.

The bis-urea-functionalized pillar[5]arene (**DUP**) mol­ecules crystallize in the monoclinic crystal system, space group *P*2_1_/*c*. In the crystal structure, one mol­ecule of di­methyl­formamide (**DMF**) is encapsulated within the cavity of the pillararene, resulting in the formation of a host–guest supra­molecular inclusion complex. As anti­cipated, the structure of the pillararene is a penta­gonal-shaped macrocycle having benzyl urea substitution at both ends of the rim. The crystal structure also reveals that one of the urea substituents is oriented above the pillar[5]arene where its N—H groups are situated just above the cavity of the macrocycle and the other urea moiety is projected outwards from the pillar[5]arene ring, as depicted in Fig. 1 ▸. In this crystal, both urea-substituted arms of the pillar[5]arene were found to be disordered and this disorder was treated specially during data refinement by applying appropriate restraints. It can be seen that the guest **DMF** mol­ecule engages in multiple inter­molecular inter­actions with pillar[5]arene ring *via* N—H⋯O or C—H⋯π inter­actions, as given in Fig. 2 ▸ and Table 1 ▸ (π being the centroids of the pillar[5]arene-based C8–C13 and C29–C34 phenyl rings). The orientation of the substituted urea arm above the pillar[5]arene cavity clearly promoted pillar[5]arene–guest inter­actions by enabling a strong N—H⋯O hydrogen bond, as depicted in Fig. 2 ▸. Such a spatial orientation of the urea spacer and subsequent N—H-mediated inter­action with the guest mol­ecule suggests the ability of these urea-substituted pillar[5]arenes to facilitate selective encapsulation and provide stable host–guest systems in a variety of applications.

## 3. Supra­molecular features

**Figure 3:**
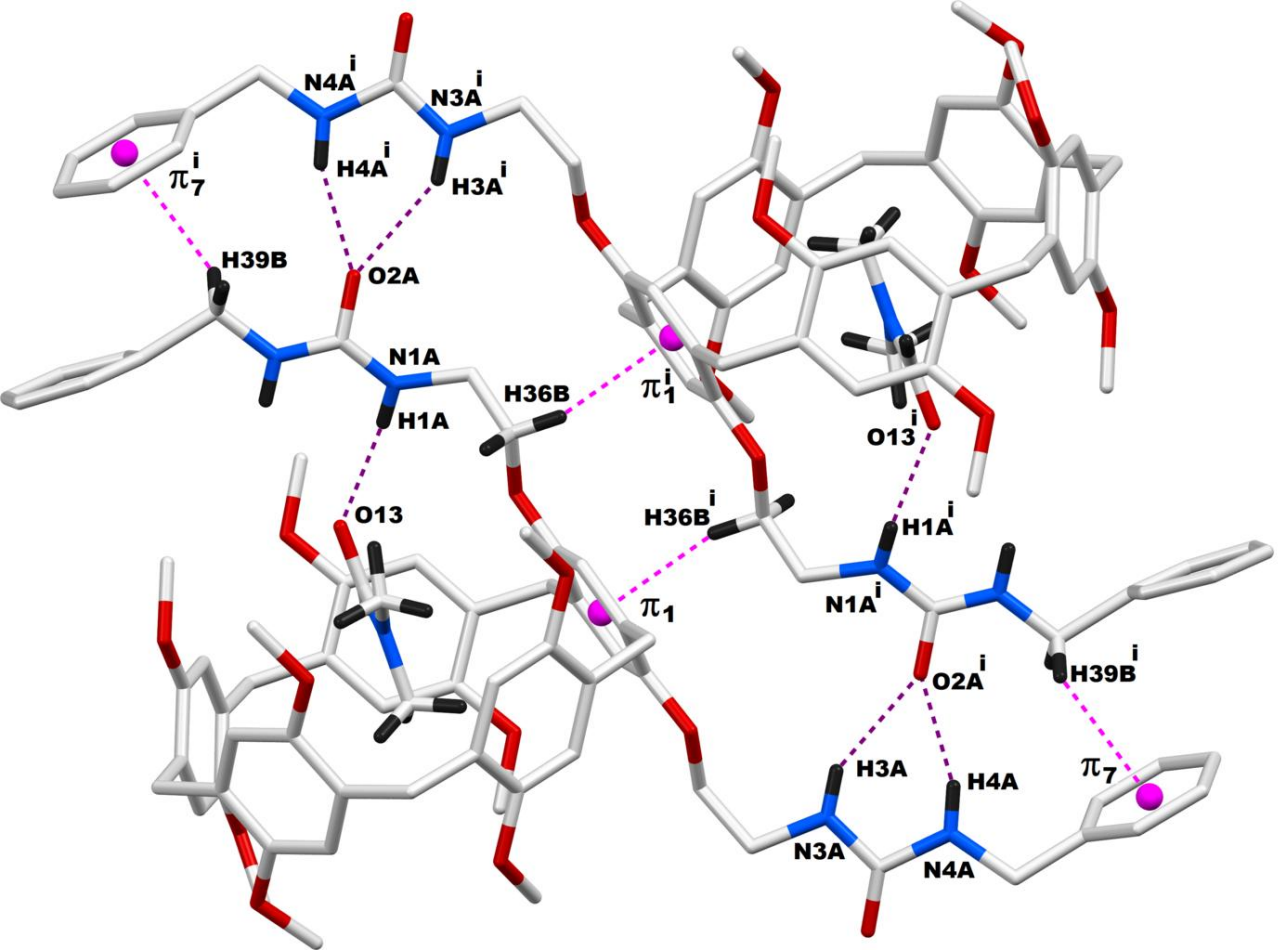
Dimer formation of the **DUB·DMF** system in the crystal through urea spacers. Symmetry code: (i) 1 − *x*, 1 − *y*, 1 − *z*; π1 and π7 are the centroids of the C1–C6 and C50*A*–C55*A* phenyl rings, respectively. Non-inter­acting hydrogen atoms on the pillar[5]arenes are omitted for clarity.

**Figure 4:**
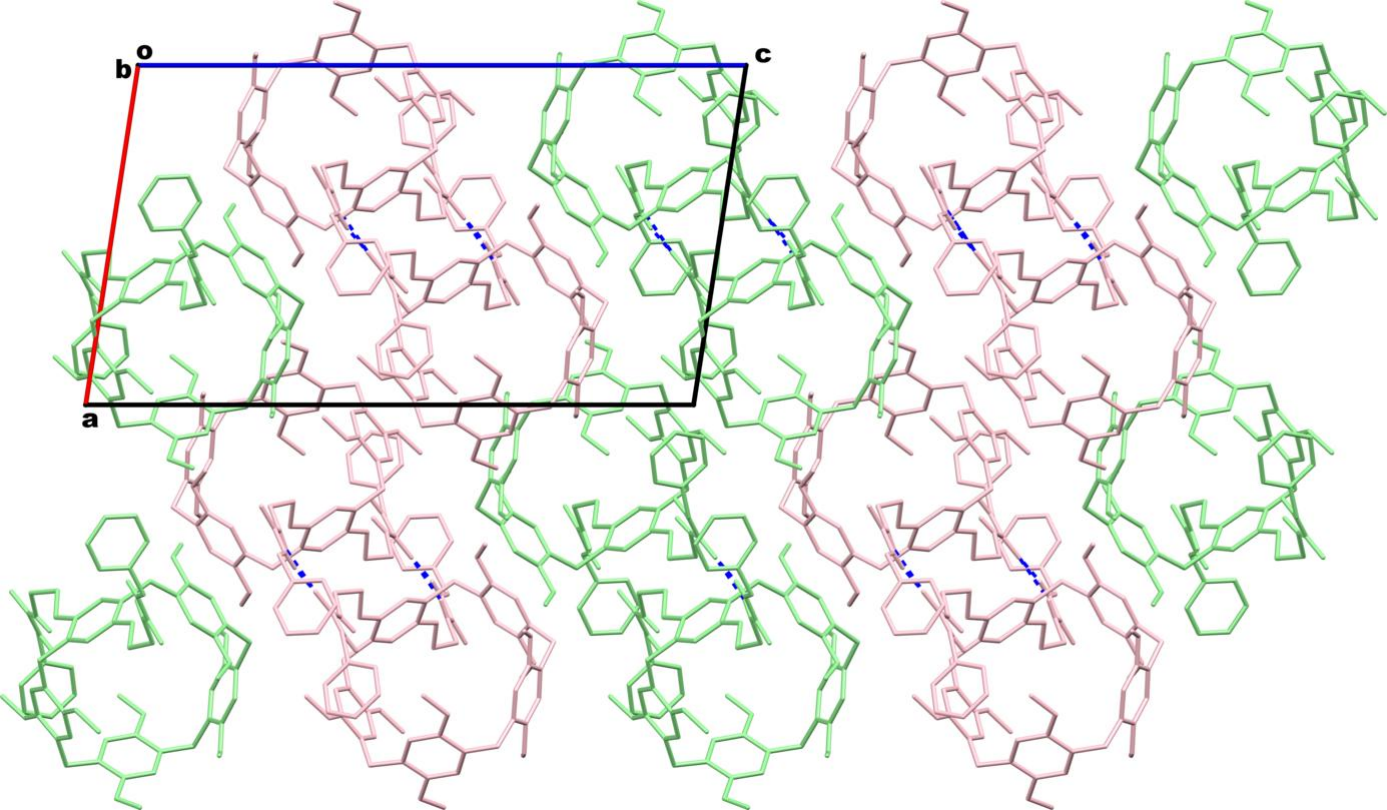
Packing pattern of **DUB** mol­ecules in the crystal. Hydrogen atoms except those on the urea moieties are omitted for clarity.

The **DUP** species are capable of involving multiple inter­molecular inter­actions in their crystal network. The qu­anti­tative details of these inter­molecular inter­actions are provided in Table 2 ▸. The multiple inter­molecular inter­actions between two adjacent pillara[5]renes are so efficient that a supra­molecular dimer is formed in this crystal system by mutual inter­actions of their urea spacers. As depicted in Fig. 3 ▸, this supra­molecular dimer is formed mainly by N—H⋯O=C inter­actions between two neighboring pillararenes. The urea N—H bonds in one arm of the pillar[5]arene are bound to the carbonyl C=O group belonging to the urea arm of a second pillar[5]arene. Furthermore, the C=O component of the other urea arm of this first pillararene is bound to the N—H groups of the second urea arm of the latter pillararene. Thus, supra­molecular dimers are produced as a result of these complementary contacts between two pillar[5]arene urea arms. Overall, in the bis-urea-pillar[5]arene system, two urea N—H groups in each pillar[5]arene are involved in supra­molecular dimer formation, and another N—H from the same pillar[5]arene is involved in supra­molecular host–guest inter­action with the DMF mol­ecule, as is evident in Fig. 3 ▸. In addition to these dimeric inter­actions, there are a few other non-bonding inter­actions between adjacent pillar[5]arenes whose qu­anti­tative details are provided in Table 2 ▸. It is observed that each pillar[5]arene unit inter­acts with four neighboring pillar[5]arenes. The packing pattern of the **DUP** mol­ecules when viewed along the *b*-axis direction is depicted in Fig. 4 ▸. The urea-based N—H⋯O hydrogen bonds through which the dimer formation occurred are also shown in this figure as blue dotted lines. This packing diagram shows sets of dimeric pillar[5]arenes propagated along the *a*-axis direction. However, the pillar[5]arenes are oriented in two different directions, which are almost perpendicular, as represented in green and pink colors.

## 4. Database survey

A search in the Cambridge Structural Database (version 5.44, last update September 2023; Groom *et al.*, 2016 ▸) reveals that no A1/A2-functionalized pillar[5]arenes substituted by benzyl urea have been reported. The crystal structure of an A1/A2- functionalized pillar[5]arene that is substituted with two urea moieties has been reported earlier (DALGOP; Cheng *et al.*, 2016 ▸). However, both urea fractions of this mol­ecule are connected to each other by a hexyl spacer, thereby making this system a mechanically self-locked pseudo[1]catenane. Similar types of mechanically self-locked pseudo[1]catenanes based on A1/A2-bis-amide-functionalized pillar[5]arenes have been reported. In these systems, the amide moieties are linked together either by *n*-alkyl spacers (HUKREM and HUKRIQ; Li *et al.*, 2015 ▸ and LIQHOM; Lv *et al.*, 2023 ▸) or by aliphatic chains containing NH, NH_2_^+^ or O heteroatoms (GACCUM, GACDAT, GACCIA and GACDEX; Liang *et al.*, 2020 ▸; LIQJOO and LIQJUU; Lv *et al.*, 2023 ▸). An A1/A2- bis-amide-functionalized pillar[5]arene cryptand with two different cavities has also been reported (MUCGIC; Wang *et al.*, 2015 ▸). Other structurally related pillararene crystals reported include an A1/A2-bis-imidazolium-functionalized pillar[5]arene (QONPEQ; Gao *et al.*, 2014 ▸), an A1/A2-bis-*N*-(9-anthrylmethy)triazole-functionalized pillar[5]arene (QACFEI; Bi *et al.*, 2016 ▸) and an A1/A2-bis-2-azido­eth­oxy-functionalized pillar[5]arene (KEWLIL; Vinodh *et al.*, 2023 ▸). Crystal structures of isomeric A1/A2, B1/B2-tetra­kis-2-azido­eth­oxy-functionalized pillar[5]arene and A1/A2, C1/C2-tetra­kis-2-azido­eth­oxy functionalized pillar[5]arene have also been reported (KEWLEH and KEWLOR; Vinodh *et al.*, 2023 ▸). Crystal structures of per-functionalized pillararenes in which all ten functionalization sites are substituted with *N*-phenyl triazole (CECDAR; Deng *et al.*, 2012 ▸), *N*-(naphthalen-2-yl-meth­yl)trizole (ACIYOC: Yu *et al.*, 2012 ▸) or phthalimide (QUYCOF; Yuan, 2020 ▸) have also been reported. The crystal structures of 4,9,14,19,24,26,28,30,32,34-deca­kis­[2-(morph­olin-4-yl)eth­oxy]pillar[5]arene in which pillararene is functionalized with ten morpholine fragments (CIZFID; Xia *et al.*, 2018 ▸) and that of 4,8,14,18,23,26,28,31,32,35-deca-[2-(pyrrol­idin-1-yl)eth­oxy]pillar[5]arene in which in which pillararene is functionalized with ten pyrrolidine fragments at their periphery (JAPGAM; Shurpik *et al.*, 2021 ▸) have also been reported in the literature. Another structurally related macrocycle reported is 5,11,17,23,29-31,32,33,34,35-deca­kis­{2-[2-(4-*t*-butyl­benzo­yl)hydrazin­yl]-2-oxoeth­oxy}calix(5)arene trideca­hydrate (KUYFAN; Hu *et al.*, 2012 ▸).

## 5. Synthesis and crystallization

The synthesis and characterization of **DUP** have been described earlier (Al-Azemi *et al.*, 2019 ▸; Vinodh *et al.*, 2023 ▸). The first step is the synthesis of A1/A2-di­bromo­eth­oxy-pillar[5]arene by the co-condensation method. The bromo-functionalized pillar[5]arene is then converted to amino derivatives by the reaction with sodium azide followed by catalytic hydrogenation. The bis-urea-functionalized pillar[5]arene **DUP** is finally synthesized upon its reaction with *p*-nitro­phenyl benzyl­carbamate. Colorless blocks of **DUP·DMF** crystals suitable for single-crystal analysis were grown by dissolving **DUP** (20mg) in DMF (0.5 mL) and keeping the solution in a 1 ml vial for 1 month.

## 6. Refinement

Crystal data, data collection and structure refinement details are summarized in Table 3 ▸. Both urea-substituted spacers (C37–C45 and C47–C55) of the pillar[5]arene in **DUP·DMF** were found to be disordered and hence the refinement of the disordered fractions was done using the PART command. The final most satisfactory occupancies for the C37–C45-urea fraction are 0.55:0.45 for the major and minor components. In the case of the C47–C55 urea fraction, the final occupancies are 0.52:0.48 for the major and minor components. In this study, only the primary components of the disordered urea moieties were taken into account to calculate the inter­molecular inter­actions (as given in Tables 1 ▸ and 2 ▸) as well as to generate Figs. 2 ▸–4 ▸ ▸. The DFIX command was used to restrain the C=O distances in the carbonyl groups of the urea fractions to 1.2 Å. In addition, the AFIX 66 command was applied to the C40*B*–C45*B* and C50*A*–C55*A* phenyl rings. In addition, DFIX commands were applied to the disordered atoms C40*A*–C45*A* and C50*B*–C55*B* to fix their bond lengths to 1.395 Å. Furthermore, DELU and SIMU commands were used in the refinement to restrain the thermal factors of the disordered C37*A* to C45*B* as well as C47*A* to C55*B* components. All the hydrogen atoms were positioned geometrically with C—H distances for methyl, methyl­ene, aromatic groups being 0.96, 0.97 and 0.93 Å, respectively, and refined with *U*_iso_(H) = 1.2*U*_eq_(C). The N—H distances were restrained to be 0.86 Å with *U*_iso_(H) = 1.2*U*_eq_(N).

## Supplementary Material

Crystal structure: contains datablock(s) I. DOI: 10.1107/S2056989023009003/jq2032sup1.cif

Structure factors: contains datablock(s) I. DOI: 10.1107/S2056989023009003/jq2032Isup3.hkl

Supporting information file. DOI: 10.1107/S2056989023009003/jq2032Isup4.cdx

CCDC reference: 2285344

Additional supporting information:  crystallographic information; 3D view; checkCIF report

## Figures and Tables

**Table 1:** Inter­molecular inter­actions(Å, °) between the pillararene host and the DMF guest π2 and π5 are the centroids of the C8–C13 and C29–C34 phenyl rings, respectively.

*D*—H⋯*A*	*D*—H	H⋯*A*	*D*⋯*A*	*D*—H⋯*A*
N1*A*—H1*A*⋯O13	0.86	2.42	2.905 (12)	116
C66—H66⋯π2	0.930	2.559	3.464	165
C64—H64*B*⋯π5	0.960	3.025	3.898	152

**Table 2:** Inter­molecular inter­actions (Å,°) engaged by DUP in the crystal network π1, π3 and π7 are the centroids of the C1–C6, C15–C20 and C51*A*–C55*A* phenyl rings, respectively.

*D*—H⋯*A*	*D*—H	H⋯*A*	*D*⋯*A*	*D*—H⋯*A*
N3*A*—H3*A*⋯O2*A* ^i^	0.86	2.35	3.13 (2)	151
N4*A*—H4*A*⋯O2*A* ^i^	0.86	2.15	2.97 (2)	159
C36—H36*B*⋯π1^i^	0.97	2.54	3.427 (3)	152
C39*A*—H39*B*⋯π7^i^	0.97	3.04	3.374 (10)	105
C43*A*—H43*A*⋯O5^ii^	0.93	2.68	3.54 (1)	155
C58—H58*A*⋯O4*A* ^iii^	0.96	2.47	3.43 (2)	172
C63—H63*C*⋯π3^iv^	0.96	2.91	3.638 (5)	133

Symmetry codes: (i) 1 − *x*, 1 − *y*, 1 − *z*; (ii) 2 − *x*, −*y*, 1 − *z*; (iii) *x*, −1 + *y*, *z*; (iv) 2 − *x*, 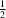 + *y*, 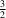 − *z*.

**Table 3:** Experimental details

Crystal data	
Chemical formula	C_63_H_70_N_4_O_12_·C_3_H_7_NO
*M*_r_	1148.32
Crystal system, space group	Monoclinic, *P*2_1_/*c*
Temperature (K)	150
*a*, *b*, *c* (Å)	14.7726 (9), 16.2952 (11), 26.1406 (15)
β (°)	98.791 (7)
*V* (Å^3^)	6218.7 (7)
*Z*	4
Radiation type	Mo *K*α
μ (mm^−1^)	0.09
Crystal size (mm)	0.20 × 0.18 × 0.11

Data collection	
Diffractometer	Rigaku R-AXIS RAPID
Absorption correction	Multi-scan (*ABSCOR*; Higashi, 1995 ▸)
*T*_min_, *T*_max_	0.652, 0.984
No. of measured, independent and observed [*I* > 2σ(*I*)] reflections	48935, 10890, 5143
*R*_int_	0.071
(sin θ/λ)_max_ (Å^−1^)	0.595

Refinement	
*R*[*F* ^2^ > 2σ(*F* ^2^)], *wR*(*F* ^2^), *S*	0.053, 0.157, 0.93
No. of reflections	10890
No. of parameters	961
No. of restraints	886
H-atom treatment	H-atom parameters constrained
Δρ_max_, Δρ_min_ (e Å^−3^)	0.19, −0.20

Computer programs: *CrystalClear* 2.1 b46 (Rigaku, 2016 ▸), *CrystalClear* 2.1 b46, *CrystalStructure* 4.2 (Rigaku, 2017 ▸), *SHELXL2019/2* (Sheldrick, 2015 ▸), *Mercury* (Macrae *et al.*, 2020 ▸).

## supplementary crystallographic information

### Crystal data

**Table:**

C_63_H_70_N_4_O_12_·C_3_H_7_NO	*F*(000) = 2448
*M**_r_* = 1148.32	*D*_x_ = 1.227 Mg m^−^^3^
Monoclinic, *P*2_1_/*c*	Mo *K*α radiation, λ = 0.71075 Å
*a* = 14.7726 (9) Å	Cell parameters from 22911 reflections
*b* = 16.2952 (11) Å	θ = 3.0–25.0°
*c* = 26.1406 (15) Å	µ = 0.09 mm^−^^1^
β = 98.791 (7)°	*T* = 150 K
*V* = 6218.7 (7) Å^3^	Block, colorless
*Z* = 4	0.20 × 0.18 × 0.11 mm

### Data collection

**Table:**

Rigaku R-AXIS RAPID diffractometer	5143 reflections with *I* > 2σ(*I*)
Detector resolution: 10.000 pixels mm^-1^	*R*_int_ = 0.071
ω scans	θ_max_ = 25.0°, θ_min_ = 3.0°
Absorption correction: multi-scan (ABSCOR; Higashi, 1995)	*h* = −17→17
*T*_min_ = 0.652, *T*_max_ = 0.984	*k* = −19→19
48935 measured reflections	*l* = −27→31
10890 independent reflections	

### Refinement

**Table:**

Refinement on *F*^2^	886 restraints
Least-squares matrix: full	Hydrogen site location: inferred from neighbouring sites
*R*[*F*^2^ > 2σ(*F*^2^)] = 0.053	H-atom parameters constrained
*wR*(*F*^2^) = 0.157	*w* = 1/[σ^2^(*F*_o_^2^) + (0.0817*P*)^2^] where *P* = (*F*_o_^2^ + 2*F*_c_^2^)/3
*S* = 0.93	(Δ/σ)_max_ < 0.001
10890 reflections	Δρ_max_ = 0.19 e Å^−^^3^
961 parameters	Δρ_min_ = −0.20 e Å^−^^3^

### Special details

**Table:**

Geometry. All esds (except the esd in the dihedral angle between two l.s. planes) are estimated using the full covariance matrix. The cell esds are taken into account individually in the estimation of esds in distances, angles and torsion angles; correlations between esds in cell parameters are only used when they are defined by crystal symmetry. An approximate (isotropic) treatment of cell esds is used for estimating esds involving l.s. planes.
Refinement. The single crystal data collection were made on Rigaku Rapid II diffractometer by Mo-Kα radiation at 150K. The data were processed by 'Crystalclear' software package. The structures were then solved by direct methods by 'CrystalStructure' crystallographic software package and the refinement was performed using SHELXL-2019/2.

### Fractional atomic coordinates and isotropic or equivalent isotropic displacement parameters (Å^2^)

**Table:**

	*x*	*y*	*z*	*U*_iso_*/*U*_eq_	Occ. (<1)
O1	0.62562 (11)	0.41032 (11)	0.50482 (6)	0.0734 (5)	
O3	0.64175 (12)	0.68117 (12)	0.63257 (7)	0.0786 (5)	
O5	0.92184 (14)	0.26904 (13)	0.48061 (7)	0.0948 (6)	
O6	0.89860 (13)	0.57968 (13)	0.56136 (7)	0.0863 (6)	
O7	0.91160 (15)	0.13445 (14)	0.67409 (8)	0.1000 (7)	
O8	1.15962 (15)	0.37805 (17)	0.65071 (9)	0.1176 (8)	
O9	0.68083 (17)	0.21549 (17)	0.79044 (10)	0.1156 (8)	
O10	1.02487 (16)	0.35280 (19)	0.80979 (8)	0.1112 (7)	
O11	0.44862 (17)	0.41066 (17)	0.68237 (9)	0.1215 (8)	
O12	0.76036 (16)	0.52578 (16)	0.80306 (9)	0.1159 (8)	
O13	0.74097 (16)	0.25255 (15)	0.56713 (8)	0.1081 (8)	
N5	0.75554 (15)	0.33315 (16)	0.63726 (9)	0.0810 (7)	
C1	0.57978 (16)	0.55146 (17)	0.60873 (9)	0.0639 (7)	
C2	0.57428 (16)	0.48341 (16)	0.57642 (10)	0.0661 (7)	
H2A	0.532823	0.441913	0.580616	0.079*	
C3	0.62917 (17)	0.47575 (16)	0.53798 (9)	0.0612 (6)	
C4	0.69112 (16)	0.53707 (16)	0.53078 (9)	0.0603 (6)	
C5	0.69554 (17)	0.60533 (16)	0.56272 (9)	0.0660 (7)	
H5	0.736165	0.647381	0.558242	0.079*	
C6	0.64089 (17)	0.61253 (16)	0.60121 (9)	0.0634 (7)	
C7	0.75205 (17)	0.53017 (17)	0.48923 (9)	0.0693 (7)	
H7A	0.716470	0.508280	0.457944	0.083*	
H7B	0.772919	0.584520	0.481368	0.083*	
C8	0.83418 (17)	0.47580 (17)	0.50520 (9)	0.0616 (7)	
C9	0.84070 (18)	0.39889 (17)	0.48388 (9)	0.0665 (7)	
H9	0.794481	0.381279	0.458008	0.080*	
C10	0.91391 (19)	0.34721 (18)	0.49985 (9)	0.0660 (7)	
C11	0.98303 (17)	0.37151 (18)	0.53847 (9)	0.0655 (7)	
C12	0.97783 (18)	0.44915 (19)	0.55917 (9)	0.0680 (7)	
H12	1.024485	0.466879	0.584758	0.082*	
C13	0.90563 (19)	0.50083 (18)	0.54299 (9)	0.0656 (7)	
C14	1.06115 (18)	0.31531 (19)	0.55950 (9)	0.0799 (8)	
H14A	1.118560	0.344909	0.561543	0.096*	
H14B	1.063027	0.269416	0.536053	0.096*	
C15	1.05064 (17)	0.28325 (18)	0.61285 (10)	0.0693 (7)	
C16	0.98837 (18)	0.22190 (18)	0.61825 (10)	0.0727 (7)	
H16	0.956062	0.197826	0.588719	0.087*	
C17	0.97263 (18)	0.19516 (18)	0.66647 (11)	0.0728 (7)	
C18	1.02084 (19)	0.22880 (19)	0.71111 (10)	0.0732 (8)	
C19	1.08329 (19)	0.2895 (2)	0.70569 (11)	0.0814 (8)	
H19	1.116930	0.312451	0.735214	0.098*	
C20	1.09758 (18)	0.31759 (19)	0.65755 (11)	0.0776 (8)	
C21	1.0057 (2)	0.1993 (2)	0.76424 (10)	0.0915 (9)	
H21A	0.993785	0.140727	0.762725	0.110*	
H21B	1.061201	0.208284	0.788635	0.110*	
C22	0.9268 (2)	0.2423 (2)	0.78379 (10)	0.0813 (9)	
C23	0.8408 (2)	0.2063 (2)	0.77991 (11)	0.0893 (9)	
H23	0.832105	0.153360	0.766719	0.107*	
C24	0.7680 (3)	0.2478 (2)	0.79535 (11)	0.0866 (9)	
C25	0.7789 (2)	0.3260 (2)	0.81507 (10)	0.0811 (8)	
C26	0.8647 (2)	0.3610 (2)	0.82018 (10)	0.0871 (9)	
H26	0.873685	0.413270	0.834303	0.105*	
C27	0.9374 (2)	0.3198 (2)	0.80469 (10)	0.0851 (9)	
C28	0.6985 (2)	0.3736 (2)	0.83055 (10)	0.0942 (10)	
H28A	0.656090	0.335489	0.842674	0.113*	
H28B	0.720914	0.410320	0.858906	0.113*	
C29	0.6482 (2)	0.4234 (2)	0.78584 (10)	0.0780 (8)	
C30	0.5692 (2)	0.3955 (2)	0.75650 (12)	0.0846 (9)	
H30	0.543575	0.346554	0.765620	0.102*	
C31	0.5268 (2)	0.4382 (2)	0.71374 (12)	0.0811 (8)	
C32	0.56182 (19)	0.51127 (19)	0.69913 (10)	0.0731 (8)	
C33	0.6401 (2)	0.5410 (2)	0.72939 (12)	0.0821 (8)	
H33	0.664376	0.590944	0.720923	0.099*	
C34	0.6826 (2)	0.4982 (2)	0.77175 (12)	0.0835 (9)	
C35	0.51981 (18)	0.55673 (19)	0.65097 (10)	0.0807 (8)	
H35A	0.511337	0.613877	0.659482	0.097*	
H35B	0.460079	0.533619	0.638298	0.097*	
C36	0.54778 (18)	0.35731 (17)	0.50083 (11)	0.0768 (8)	
H36A	0.546859	0.329834	0.533660	0.092*	0.549 (9)
H36B	0.492174	0.389519	0.492993	0.092*	0.549 (9)
H36C	0.491123	0.388230	0.493938	0.092*	0.451 (9)
H36D	0.548120	0.325478	0.532211	0.092*	0.451 (9)
C38A	0.6146 (11)	0.1591 (14)	0.4628 (5)	0.0720 (19)	0.549 (9)
O2A	0.5517 (11)	0.1335 (13)	0.4324 (6)	0.097 (3)	0.549 (9)
C37A	0.5507 (13)	0.2980 (14)	0.4614 (6)	0.076 (2)	0.549 (9)
H37A	0.553962	0.327252	0.429441	0.091*	0.549 (9)
H37B	0.492864	0.268707	0.456790	0.091*	0.549 (9)
N1A	0.6231 (11)	0.2377 (11)	0.4679 (4)	0.071 (2)	0.549 (9)
H1A	0.677988	0.256062	0.476067	0.086*	0.549 (9)
N2A	0.6760 (8)	0.1091 (8)	0.4760 (3)	0.0854 (18)	0.549 (9)
H2A1	0.726736	0.128459	0.491963	0.103*	0.549 (9)
C39A	0.6727 (6)	0.0255 (6)	0.4680 (4)	0.110 (2)	0.549 (9)
H39A	0.671104	0.014088	0.431415	0.133*	0.549 (9)
H39B	0.617232	0.003560	0.478351	0.133*	0.549 (9)
C40A	0.7612 (7)	−0.0185 (7)	0.5009 (4)	0.1066 (16)	0.549 (9)
C41A	0.7884 (7)	−0.0694 (7)	0.5421 (4)	0.1199 (18)	0.549 (9)
H41A	0.750734	−0.075098	0.567290	0.144*	0.549 (9)
C42A	0.8678 (8)	−0.1116 (7)	0.5474 (4)	0.129 (2)	0.549 (9)
H42A	0.884325	−0.146034	0.575608	0.155*	0.549 (9)
C43A	0.9218 (7)	−0.1032 (7)	0.5116 (5)	0.128 (2)	0.549 (9)
H43A	0.976352	−0.132549	0.514978	0.153*	0.549 (9)
C44A	0.8988 (7)	−0.0517 (7)	0.4692 (4)	0.137 (2)	0.549 (9)
H44A	0.936229	−0.047514	0.443779	0.164*	0.549 (9)
C45A	0.8189 (7)	−0.0069 (7)	0.4660 (4)	0.125 (2)	0.549 (9)
H45A	0.804667	0.031552	0.439746	0.150*	0.549 (9)
O2B	0.5485 (12)	0.1386 (16)	0.4186 (7)	0.083 (3)	0.451 (9)
C38B	0.6085 (15)	0.1585 (18)	0.4526 (6)	0.075 (2)	0.451 (9)
C37B	0.5607 (16)	0.2987 (17)	0.4522 (8)	0.078 (3)	0.451 (9)
H37C	0.503243	0.274214	0.436518	0.094*	0.451 (9)
H37D	0.587969	0.327971	0.426071	0.094*	0.451 (9)
N1B	0.6252 (14)	0.2360 (13)	0.4800 (6)	0.080 (2)	0.451 (9)
H1B	0.664819	0.244481	0.507159	0.095*	0.451 (9)
N2B	0.6821 (10)	0.1073 (10)	0.4948 (4)	0.093 (2)	0.451 (9)
H2B	0.725341	0.131486	0.515076	0.112*	0.451 (9)
C39B	0.6696 (7)	0.0226 (7)	0.4956 (5)	0.108 (2)	0.451 (9)
H39C	0.626055	0.005815	0.465855	0.130*	0.451 (9)
H39D	0.645533	0.006836	0.526695	0.130*	0.451 (9)
C40B	0.7600 (7)	−0.0189 (8)	0.4944 (4)	0.1065 (18)	0.451 (9)
C41B	0.7598 (6)	−0.0549 (7)	0.5426 (4)	0.115 (2)	0.451 (9)
H41B	0.710659	−0.046639	0.560350	0.138*	0.451 (9)
C42B	0.8332 (7)	−0.1034 (6)	0.5642 (3)	0.124 (2)	0.451 (9)
H42B	0.833168	−0.127548	0.596458	0.149*	0.451 (9)
C43B	0.9068 (6)	−0.1158 (6)	0.5377 (5)	0.126 (2)	0.451 (9)
H43B	0.955877	−0.148252	0.552160	0.151*	0.451 (9)
C44B	0.9069 (6)	−0.0797 (7)	0.4895 (5)	0.130 (2)	0.451 (9)
H44B	0.956076	−0.088048	0.471752	0.157*	0.451 (9)
C45B	0.8335 (8)	−0.0313 (7)	0.4679 (3)	0.121 (2)	0.451 (9)
H45B	0.833567	−0.007138	0.435642	0.146*	0.451 (9)
C46	0.7154 (2)	0.73728 (19)	0.63378 (12)	0.0901 (9)	
H46A	0.717782	0.755680	0.598722	0.108*	0.525 (6)
H46B	0.772107	0.708492	0.645800	0.108*	0.525 (6)
H46C	0.716224	0.762694	0.600299	0.108*	0.475 (6)
H46D	0.774003	0.711325	0.645381	0.108*	0.475 (6)
O4A	0.7106 (11)	0.9714 (15)	0.6819 (8)	0.108 (4)	0.525 (6)
C48A	0.6429 (11)	0.9419 (16)	0.6576 (9)	0.084 (2)	0.525 (6)
C47A	0.7103 (10)	0.8110 (11)	0.6673 (7)	0.087 (3)	0.525 (6)
H47A	0.700703	0.794037	0.701595	0.105*	0.525 (6)
H47B	0.767259	0.841464	0.670413	0.105*	0.525 (6)
N3A	0.6335 (13)	0.8633 (11)	0.6436 (5)	0.084 (2)	0.525 (6)
H3A	0.587273	0.844836	0.622787	0.100*	0.525 (6)
N4A	0.5565 (11)	0.9843 (13)	0.6390 (5)	0.090 (2)	0.525 (6)
H4A	0.514585	0.959867	0.617957	0.108*	0.525 (6)
C49A	0.5415 (5)	1.0667 (4)	0.6559 (3)	0.1006 (17)	0.525 (6)
H49A	0.599252	1.096144	0.660775	0.121*	0.525 (6)
H49B	0.519401	1.064660	0.688912	0.121*	0.525 (6)
C50A	0.4724 (5)	1.1128 (5)	0.6169 (3)	0.1028 (18)	0.525 (6)
C51A	0.4855 (4)	1.1273 (5)	0.5661 (3)	0.127 (2)	0.525 (6)
H51A	0.538237	1.108486	0.554517	0.153*	0.525 (6)
C52A	0.4197 (5)	1.1699 (5)	0.5326 (2)	0.138 (2)	0.525 (6)
H52A	0.428396	1.179572	0.498663	0.166*	0.525 (6)
C53A	0.3408 (5)	1.1979 (5)	0.5499 (3)	0.140 (2)	0.525 (6)
H53A	0.296765	1.226434	0.527532	0.168*	0.525 (6)
C54A	0.3277 (4)	1.1834 (5)	0.6007 (3)	0.131 (2)	0.525 (6)
H54A	0.274974	1.202208	0.612255	0.157*	0.525 (6)
C55A	0.3935 (5)	1.1408 (5)	0.6341 (2)	0.124 (2)	0.525 (6)
H55A	0.384814	1.131122	0.668110	0.149*	0.525 (6)
O4B	0.6891 (12)	0.9712 (16)	0.6886 (8)	0.102 (4)	0.475 (6)
C48B	0.6277 (13)	0.9417 (17)	0.6589 (10)	0.080 (2)	0.475 (6)
C47B	0.6902 (11)	0.7989 (12)	0.6736 (7)	0.087 (3)	0.475 (6)
H47C	0.669108	0.768609	0.701451	0.105*	0.475 (6)
H47D	0.745209	0.828264	0.688229	0.105*	0.475 (6)
N3B	0.6230 (15)	0.8563 (12)	0.6544 (6)	0.081 (2)	0.475 (6)
H3B	0.572833	0.836662	0.637991	0.097*	0.475 (6)
N4B	0.5715 (12)	0.9870 (14)	0.6288 (6)	0.091 (2)	0.475 (6)
H4B	0.527426	0.963550	0.608803	0.109*	0.475 (6)
C49B	0.5786 (6)	1.0744 (5)	0.6271 (4)	0.1059 (19)	0.475 (6)
H49C	0.622152	1.088971	0.604343	0.127*	0.475 (6)
H49D	0.602335	1.094390	0.661430	0.127*	0.475 (6)
C50B	0.4904 (7)	1.1158 (8)	0.6087 (4)	0.1045 (18)	0.475 (6)
C51B	0.4736 (7)	1.1659 (6)	0.5671 (4)	0.116 (2)	0.475 (6)
H51B	0.521468	1.173736	0.548265	0.140*	0.475 (6)
C52B	0.3946 (8)	1.2055 (7)	0.5499 (4)	0.127 (2)	0.475 (6)
H52B	0.389372	1.238008	0.520346	0.152*	0.475 (6)
C53B	0.3263 (9)	1.1979 (8)	0.5751 (5)	0.135 (2)	0.475 (6)
H53B	0.272619	1.227287	0.565003	0.161*	0.475 (6)
C54B	0.3335 (9)	1.1457 (8)	0.6172 (4)	0.135 (2)	0.475 (6)
H54B	0.282623	1.135820	0.633331	0.162*	0.475 (6)
C55B	0.4189 (8)	1.1070 (7)	0.6360 (4)	0.122 (2)	0.475 (6)
H55B	0.425805	1.076300	0.666334	0.146*	0.475 (6)
C56	0.8593 (2)	0.2434 (2)	0.43776 (13)	0.1086 (11)	
H56A	0.874983	0.189156	0.427819	0.130*	
H56B	0.861423	0.280506	0.409431	0.130*	
H56C	0.798617	0.243064	0.446685	0.130*	
C57	0.9618 (2)	0.6051 (2)	0.60406 (12)	0.1104 (11)	
H57A	0.948198	0.660376	0.613092	0.132*	
H57B	1.022619	0.602836	0.595385	0.132*	
H57C	0.957892	0.569530	0.632907	0.132*	
C58	0.8575 (2)	0.0987 (2)	0.63023 (15)	0.1204 (13)	
H58A	0.815701	0.059906	0.641414	0.144*	
H58B	0.896623	0.071097	0.609604	0.144*	
H58C	0.823631	0.140839	0.609981	0.144*	
C59	1.1860 (3)	0.4329 (3)	0.69066 (18)	0.182 (2)	
H59A	1.132545	0.454135	0.702913	0.218*	
H59B	1.219473	0.477358	0.678326	0.218*	
H59C	1.224118	0.405403	0.718449	0.218*	
C60	0.6671 (3)	0.1327 (3)	0.77892 (18)	0.1529 (17)	
H60A	0.699176	0.100228	0.806554	0.184*	
H60B	0.689870	0.120232	0.747321	0.184*	
H60C	0.602850	0.120512	0.774942	0.184*	
C61	1.0326 (3)	0.4372 (3)	0.8090 (2)	0.1614 (19)	
H61A	1.095417	0.451999	0.808573	0.194*	
H61B	1.012638	0.459789	0.839314	0.194*	
H61C	0.995287	0.458465	0.778638	0.194*	
C62	0.4123 (3)	0.3358 (3)	0.6924 (2)	0.189 (2)	
H62A	0.362091	0.323138	0.665727	0.227*	
H62B	0.390905	0.337763	0.725264	0.227*	
H62C	0.458584	0.294267	0.693258	0.227*	
C63	0.8047 (3)	0.5954 (3)	0.78706 (18)	0.1627 (19)	
H63A	0.763236	0.641179	0.783829	0.195*	
H63B	0.823807	0.584728	0.754220	0.195*	
H63C	0.857272	0.608002	0.812217	0.195*	
C64	0.6843 (3)	0.2918 (3)	0.65897 (15)	0.1474 (16)	
H64A	0.650013	0.257569	0.633068	0.177*	
H64B	0.644259	0.331635	0.670708	0.177*	
H64C	0.711158	0.258541	0.687644	0.177*	
C65	0.8021 (3)	0.4008 (3)	0.66514 (13)	0.1316 (14)	
H65A	0.759391	0.444566	0.667369	0.158*	
H65B	0.850303	0.419692	0.647325	0.158*	
H65C	0.827572	0.383174	0.699372	0.158*	
C66	0.7751 (2)	0.3092 (2)	0.59272 (11)	0.0829 (9)	
H66	0.819847	0.338843	0.579294	0.099*	

### Atomic displacement parameters (Å^2^)

**Table:**

	*U*^11^	*U*^22^	*U*^33^	*U*^12^	*U*^13^	*U*^23^
O1	0.0728 (12)	0.0564 (12)	0.0902 (12)	−0.0020 (10)	0.0095 (9)	−0.0117 (10)
O3	0.0809 (12)	0.0637 (14)	0.0912 (12)	0.0038 (11)	0.0134 (9)	−0.0192 (10)
O5	0.1073 (15)	0.0850 (17)	0.0872 (13)	0.0167 (12)	−0.0007 (11)	−0.0277 (12)
O6	0.0845 (13)	0.0796 (16)	0.0961 (13)	−0.0122 (11)	0.0182 (10)	−0.0272 (12)
O7	0.1071 (16)	0.0854 (17)	0.1028 (15)	−0.0224 (14)	0.0012 (12)	0.0122 (12)
O8	0.1035 (16)	0.134 (2)	0.1101 (16)	−0.0527 (16)	0.0006 (13)	−0.0014 (15)
O9	0.1125 (19)	0.097 (2)	0.1443 (19)	−0.0131 (16)	0.0409 (14)	0.0048 (15)
O10	0.0924 (17)	0.125 (2)	0.1089 (16)	−0.0047 (16)	−0.0061 (12)	−0.0237 (15)
O11	0.1072 (17)	0.112 (2)	0.1376 (19)	−0.0312 (16)	−0.0078 (15)	0.0056 (16)
O12	0.1061 (17)	0.109 (2)	0.1220 (17)	−0.0249 (15)	−0.0149 (14)	0.0053 (14)
O13	0.1306 (19)	0.0932 (19)	0.0878 (14)	0.0077 (14)	−0.0241 (13)	−0.0129 (13)
N5	0.0830 (16)	0.094 (2)	0.0665 (14)	0.0043 (14)	0.0121 (12)	0.0049 (13)
C1	0.0585 (15)	0.0600 (19)	0.0726 (16)	0.0112 (14)	0.0082 (12)	0.0016 (14)
C2	0.0585 (15)	0.0569 (19)	0.0819 (17)	0.0047 (13)	0.0077 (13)	0.0061 (14)
C3	0.0646 (16)	0.0464 (18)	0.0697 (16)	0.0083 (14)	0.0011 (12)	−0.0029 (13)
C4	0.0658 (16)	0.0461 (17)	0.0670 (15)	0.0062 (14)	0.0039 (12)	0.0018 (13)
C5	0.0678 (16)	0.0528 (18)	0.0760 (16)	0.0026 (13)	0.0069 (13)	0.0041 (14)
C6	0.0643 (16)	0.0532 (19)	0.0702 (16)	0.0089 (14)	0.0024 (13)	−0.0053 (13)
C7	0.0819 (18)	0.0620 (19)	0.0654 (15)	−0.0013 (15)	0.0156 (13)	0.0032 (13)
C8	0.0686 (16)	0.066 (2)	0.0519 (13)	−0.0036 (15)	0.0159 (12)	−0.0018 (13)
C9	0.0747 (18)	0.072 (2)	0.0534 (14)	−0.0020 (16)	0.0098 (12)	−0.0107 (14)
C10	0.0786 (18)	0.064 (2)	0.0574 (15)	0.0018 (16)	0.0154 (13)	−0.0085 (14)
C11	0.0630 (16)	0.081 (2)	0.0547 (14)	0.0018 (15)	0.0160 (12)	−0.0013 (14)
C12	0.0653 (17)	0.083 (2)	0.0568 (15)	−0.0124 (16)	0.0141 (12)	−0.0117 (15)
C13	0.0723 (18)	0.064 (2)	0.0650 (15)	−0.0089 (16)	0.0243 (14)	−0.0111 (14)
C14	0.0689 (17)	0.099 (2)	0.0733 (17)	0.0080 (17)	0.0156 (13)	−0.0006 (16)
C15	0.0577 (16)	0.078 (2)	0.0706 (17)	0.0058 (15)	0.0028 (13)	0.0011 (14)
C16	0.0725 (18)	0.071 (2)	0.0689 (17)	0.0034 (16)	−0.0063 (13)	−0.0033 (14)
C17	0.0642 (17)	0.063 (2)	0.087 (2)	0.0017 (15)	−0.0024 (14)	0.0076 (15)
C18	0.0694 (18)	0.074 (2)	0.0723 (18)	0.0104 (16)	−0.0012 (14)	0.0105 (15)
C19	0.0767 (19)	0.089 (2)	0.0717 (18)	0.0022 (18)	−0.0116 (14)	−0.0010 (16)
C20	0.0625 (17)	0.083 (2)	0.083 (2)	−0.0093 (16)	−0.0012 (14)	−0.0014 (16)
C21	0.098 (2)	0.097 (3)	0.0741 (18)	0.020 (2)	−0.0046 (16)	0.0201 (16)
C22	0.096 (2)	0.086 (3)	0.0582 (16)	0.011 (2)	0.0009 (15)	0.0175 (16)
C23	0.106 (3)	0.078 (2)	0.084 (2)	0.003 (2)	0.0143 (18)	0.0148 (16)
C24	0.099 (3)	0.085 (3)	0.0765 (19)	−0.004 (2)	0.0163 (17)	0.0161 (18)
C25	0.100 (2)	0.089 (3)	0.0554 (15)	0.007 (2)	0.0126 (15)	0.0134 (16)
C26	0.103 (2)	0.097 (3)	0.0582 (16)	−0.003 (2)	0.0017 (15)	0.0004 (15)
C27	0.086 (2)	0.103 (3)	0.0621 (17)	0.004 (2)	−0.0031 (15)	0.0037 (17)
C28	0.110 (2)	0.107 (3)	0.0696 (18)	0.000 (2)	0.0252 (17)	0.0061 (17)
C29	0.085 (2)	0.082 (2)	0.0719 (18)	−0.0001 (18)	0.0287 (16)	−0.0066 (16)
C30	0.086 (2)	0.085 (2)	0.088 (2)	−0.0121 (19)	0.0284 (17)	0.0017 (18)
C31	0.0705 (19)	0.086 (3)	0.088 (2)	−0.0102 (18)	0.0167 (16)	−0.0110 (18)
C32	0.0691 (18)	0.074 (2)	0.0799 (18)	0.0019 (16)	0.0241 (15)	−0.0043 (16)
C33	0.0755 (19)	0.080 (2)	0.093 (2)	−0.0063 (17)	0.0188 (16)	0.0029 (17)
C34	0.077 (2)	0.090 (3)	0.084 (2)	−0.0061 (19)	0.0125 (16)	−0.0091 (18)
C35	0.0725 (17)	0.082 (2)	0.0904 (19)	0.0143 (16)	0.0227 (15)	−0.0012 (16)
C36	0.0721 (17)	0.0569 (19)	0.0954 (19)	0.0053 (15)	−0.0062 (14)	−0.0084 (15)
C38A	0.075 (3)	0.057 (3)	0.077 (4)	0.009 (3)	−0.013 (3)	−0.010 (4)
O2A	0.098 (4)	0.069 (4)	0.109 (7)	0.000 (3)	−0.027 (5)	−0.026 (6)
C37A	0.075 (4)	0.061 (3)	0.085 (5)	0.011 (3)	−0.011 (4)	−0.008 (4)
N1A	0.065 (3)	0.066 (3)	0.074 (4)	0.007 (2)	−0.023 (3)	−0.013 (4)
N2A	0.095 (3)	0.062 (3)	0.094 (4)	0.019 (2)	−0.003 (4)	0.009 (4)
C39A	0.121 (3)	0.085 (3)	0.120 (4)	0.010 (3)	0.002 (4)	−0.001 (4)
C40A	0.113 (3)	0.082 (3)	0.124 (3)	0.033 (3)	0.014 (3)	0.013 (3)
C41A	0.123 (4)	0.095 (4)	0.140 (4)	0.039 (3)	0.013 (3)	0.013 (3)
C42A	0.124 (5)	0.106 (4)	0.152 (4)	0.043 (4)	0.004 (4)	0.009 (4)
C43A	0.122 (4)	0.108 (4)	0.145 (5)	0.057 (3)	−0.002 (3)	0.011 (4)
C44A	0.131 (4)	0.117 (5)	0.157 (5)	0.056 (4)	0.008 (4)	0.015 (4)
C45A	0.122 (4)	0.106 (5)	0.145 (4)	0.042 (3)	0.017 (3)	0.017 (3)
O2B	0.074 (5)	0.079 (6)	0.092 (7)	0.000 (4)	−0.005 (4)	−0.028 (5)
C38B	0.079 (4)	0.062 (3)	0.082 (5)	0.008 (3)	0.010 (4)	−0.004 (4)
C37B	0.078 (5)	0.062 (4)	0.087 (5)	0.012 (4)	−0.012 (4)	−0.013 (4)
N1B	0.080 (3)	0.066 (3)	0.084 (6)	0.015 (3)	−0.013 (4)	−0.011 (4)
N2B	0.105 (3)	0.062 (3)	0.107 (5)	0.002 (3)	0.000 (4)	−0.007 (5)
C39B	0.118 (4)	0.082 (4)	0.121 (5)	0.016 (3)	0.008 (4)	0.015 (4)
C40B	0.113 (3)	0.084 (3)	0.122 (4)	0.029 (3)	0.016 (3)	0.010 (3)
C41B	0.116 (5)	0.096 (4)	0.132 (4)	0.032 (4)	0.016 (4)	0.012 (4)
C42B	0.120 (5)	0.102 (5)	0.145 (5)	0.028 (4)	0.005 (4)	0.014 (4)
C43B	0.117 (5)	0.107 (4)	0.150 (5)	0.044 (4)	0.006 (4)	0.015 (4)
C44B	0.129 (4)	0.110 (5)	0.150 (5)	0.047 (4)	0.011 (4)	0.019 (4)
C45B	0.121 (4)	0.103 (5)	0.140 (4)	0.046 (4)	0.017 (3)	0.015 (4)
C46	0.0774 (19)	0.077 (2)	0.111 (2)	0.0006 (17)	−0.0009 (16)	−0.0283 (17)
O4A	0.087 (6)	0.086 (5)	0.141 (7)	−0.024 (5)	−0.013 (5)	−0.009 (5)
C48A	0.087 (5)	0.067 (3)	0.097 (4)	−0.008 (4)	0.010 (4)	−0.014 (3)
C47A	0.079 (5)	0.077 (5)	0.103 (5)	−0.002 (4)	0.002 (4)	−0.021 (4)
N3A	0.076 (4)	0.071 (4)	0.099 (5)	−0.003 (3)	−0.002 (4)	−0.016 (4)
N4A	0.091 (4)	0.066 (3)	0.109 (4)	−0.010 (3)	0.004 (3)	−0.015 (4)
C49A	0.111 (4)	0.070 (3)	0.120 (4)	−0.005 (3)	0.015 (3)	−0.014 (3)
C50A	0.109 (4)	0.071 (3)	0.130 (4)	−0.014 (3)	0.020 (3)	0.005 (3)
C51A	0.127 (4)	0.110 (5)	0.148 (4)	−0.007 (4)	0.029 (3)	0.023 (4)
C52A	0.148 (5)	0.125 (5)	0.144 (4)	−0.005 (4)	0.030 (4)	0.031 (4)
C53A	0.156 (4)	0.117 (4)	0.148 (4)	0.005 (4)	0.027 (4)	0.028 (4)
C54A	0.150 (4)	0.104 (4)	0.139 (4)	0.021 (4)	0.026 (4)	0.031 (4)
C55A	0.136 (4)	0.096 (4)	0.139 (4)	0.021 (4)	0.017 (3)	0.014 (3)
O4B	0.098 (7)	0.080 (5)	0.119 (6)	−0.014 (6)	−0.017 (6)	−0.033 (5)
C48B	0.078 (4)	0.064 (3)	0.095 (4)	−0.016 (4)	0.006 (4)	−0.016 (3)
C47B	0.081 (5)	0.075 (5)	0.101 (5)	−0.006 (4)	0.000 (4)	−0.025 (4)
N3B	0.077 (4)	0.066 (3)	0.098 (5)	−0.003 (3)	0.009 (4)	−0.019 (4)
N4B	0.093 (5)	0.066 (3)	0.109 (5)	−0.014 (4)	0.000 (4)	−0.013 (4)
C49B	0.112 (4)	0.069 (3)	0.133 (4)	−0.015 (3)	0.005 (3)	0.000 (4)
C50B	0.113 (3)	0.072 (3)	0.128 (4)	−0.014 (3)	0.016 (3)	0.005 (3)
C51B	0.118 (4)	0.091 (5)	0.141 (4)	−0.004 (4)	0.024 (3)	0.016 (4)
C52B	0.129 (4)	0.105 (4)	0.148 (4)	0.006 (4)	0.029 (4)	0.025 (4)
C53B	0.149 (4)	0.111 (4)	0.146 (5)	0.015 (4)	0.031 (4)	0.019 (4)
C54B	0.147 (4)	0.111 (5)	0.153 (5)	0.000 (4)	0.041 (4)	0.016 (4)
C55B	0.139 (4)	0.090 (4)	0.139 (4)	0.004 (4)	0.032 (3)	0.011 (3)
C56	0.108 (2)	0.101 (3)	0.115 (2)	0.000 (2)	0.010 (2)	−0.046 (2)
C57	0.122 (3)	0.108 (3)	0.103 (2)	−0.026 (2)	0.020 (2)	−0.045 (2)
C58	0.111 (3)	0.087 (3)	0.148 (3)	−0.029 (2)	−0.028 (2)	0.026 (2)
C59	0.211 (5)	0.168 (5)	0.158 (4)	−0.114 (4)	−0.001 (3)	−0.024 (4)
C60	0.151 (4)	0.118 (4)	0.199 (5)	−0.038 (3)	0.058 (3)	−0.011 (3)
C61	0.112 (3)	0.134 (5)	0.236 (5)	−0.029 (3)	0.021 (3)	−0.060 (4)
C62	0.151 (4)	0.156 (5)	0.241 (6)	−0.075 (4)	−0.027 (4)	0.034 (4)
C63	0.133 (3)	0.154 (5)	0.187 (4)	−0.068 (3)	−0.021 (3)	0.030 (3)
C64	0.151 (3)	0.177 (5)	0.128 (3)	−0.022 (3)	0.065 (3)	0.020 (3)
C65	0.160 (3)	0.136 (4)	0.095 (2)	−0.009 (3)	0.007 (2)	−0.028 (2)
C66	0.083 (2)	0.102 (3)	0.0623 (18)	0.0095 (19)	0.0071 (15)	0.0061 (17)

### Geometric parameters (Å, º)

**Table:**

O1—C3	1.370 (3)	C44A—C45A	1.379 (11)
O1—C36	1.429 (3)	C44A—H44A	0.9300
O3—C6	1.386 (3)	C45A—H45A	0.9300
O3—C46	1.417 (3)	O2B—C38B	1.2008 (11)
O5—C10	1.381 (3)	C38B—N1B	1.45 (4)
O5—C56	1.402 (3)	C38B—N2B	1.65 (3)
O6—C13	1.381 (3)	C37B—N1B	1.51 (4)
O6—C57	1.404 (3)	C37B—H37C	0.9700
O7—C17	1.373 (3)	C37B—H37D	0.9700
O7—C58	1.419 (4)	N1B—H1B	0.8600
O8—C20	1.376 (3)	N2B—C39B	1.394 (16)
O8—C59	1.385 (4)	N2B—H2B	0.8600
O9—C24	1.379 (4)	C39B—C40B	1.500 (13)
O9—C60	1.390 (4)	C39B—H39C	0.9700
O10—C61	1.380 (5)	C39B—H39D	0.9700
O10—C27	1.386 (4)	C40B—C41B	1.3900
O11—C62	1.374 (4)	C40B—C45B	1.3900
O11—C31	1.385 (3)	C41B—C42B	1.3900
O12—C34	1.380 (3)	C41B—H41B	0.9300
O12—C63	1.406 (4)	C42B—C43B	1.3900
O13—C66	1.204 (3)	C42B—H42B	0.9300
N5—C66	1.301 (3)	C43B—C44B	1.3900
N5—C65	1.437 (4)	C43B—H43B	0.9300
N5—C64	1.437 (4)	C44B—C45B	1.3900
C1—C6	1.378 (3)	C44B—H44B	0.9300
C1—C2	1.389 (3)	C45B—H45B	0.9300
C1—C35	1.520 (3)	C46—C47A	1.495 (18)
C2—C3	1.390 (3)	C46—C47B	1.53 (2)
C2—H2A	0.9300	C46—H46A	0.9700
C3—C4	1.387 (3)	C46—H46B	0.9700
C4—C5	1.386 (3)	C46—H46C	0.9700
C4—C7	1.517 (3)	C46—H46D	0.9700
C5—C6	1.388 (3)	O4A—C48A	1.2008 (11)
C5—H5	0.9300	C48A—N3A	1.33 (3)
C7—C8	1.509 (3)	C48A—N4A	1.47 (3)
C7—H7A	0.9700	C47A—N3A	1.48 (2)
C7—H7B	0.9700	C47A—H47A	0.9700
C8—C9	1.381 (3)	C47A—H47B	0.9700
C8—C13	1.392 (3)	N3A—H3A	0.8600
C9—C10	1.384 (3)	N4A—C49A	1.44 (2)
C9—H9	0.9300	N4A—H4A	0.8600
C10—C11	1.380 (3)	C49A—C50A	1.526 (8)
C11—C12	1.383 (4)	C49A—H49A	0.9700
C11—C14	1.510 (3)	C49A—H49B	0.9700
C12—C13	1.374 (4)	C50A—C51A	1.3900
C12—H12	0.9300	C50A—C55A	1.3900
C14—C15	1.519 (3)	C51A—C52A	1.3900
C14—H14A	0.9700	C51A—H51A	0.9300
C14—H14B	0.9700	C52A—C53A	1.3900
C15—C16	1.380 (4)	C52A—H52A	0.9300
C15—C20	1.383 (4)	C53A—C54A	1.3900
C16—C17	1.386 (4)	C53A—H53A	0.9300
C16—H16	0.9300	C54A—C55A	1.3900
C17—C18	1.384 (4)	C54A—H54A	0.9300
C18—C19	1.375 (4)	C55A—H55A	0.9300
C18—C21	1.518 (4)	O4B—C48B	1.2008 (11)
C19—C20	1.385 (4)	C48B—N4B	1.29 (3)
C19—H19	0.9300	C48B—N3B	1.40 (3)
C21—C22	1.514 (4)	C47B—N3B	1.40 (2)
C21—H21A	0.9700	C47B—H47C	0.9700
C21—H21B	0.9700	C47B—H47D	0.9700
C22—C27	1.376 (4)	N3B—H3B	0.8600
C22—C23	1.389 (4)	N4B—C49B	1.43 (2)
C23—C24	1.383 (4)	N4B—H4B	0.8600
C23—H23	0.9300	C49B—C50B	1.481 (11)
C24—C25	1.374 (4)	C49B—H49C	0.9700
C25—C26	1.378 (4)	C49B—H49D	0.9700
C25—C28	1.524 (4)	C50B—C51B	1.352 (13)
C26—C27	1.379 (4)	C50B—C55B	1.370 (8)
C26—H26	0.9300	C51B—C52B	1.349 (12)
C28—C29	1.520 (4)	C51B—H51B	0.9300
C28—H28A	0.9700	C52B—C53B	1.293 (14)
C28—H28B	0.9700	C52B—H52B	0.9300
C29—C30	1.372 (4)	C53B—C54B	1.380 (14)
C29—C34	1.392 (4)	C53B—H53B	0.9300
C30—C31	1.383 (4)	C54B—C55B	1.429 (14)
C30—H30	0.9300	C54B—H54B	0.9300
C31—C32	1.376 (4)	C55B—H55B	0.9300
C32—C33	1.385 (4)	C56—H56A	0.9600
C32—C35	1.510 (4)	C56—H56B	0.9600
C33—C34	1.376 (4)	C56—H56C	0.9600
C33—H33	0.9300	C57—H57A	0.9600
C35—H35A	0.9700	C57—H57B	0.9600
C35—H35B	0.9700	C57—H57C	0.9600
C36—C37A	1.418 (18)	C58—H58A	0.9600
C36—C37B	1.62 (2)	C58—H58B	0.9600
C36—H36A	0.9700	C58—H58C	0.9600
C36—H36B	0.9700	C59—H59A	0.9600
C36—H36C	0.9700	C59—H59B	0.9600
C36—H36D	0.9700	C59—H59C	0.9600
C38A—O2A	1.2011 (10)	C60—H60A	0.9600
C38A—N2A	1.23 (3)	C60—H60B	0.9600
C38A—N1A	1.29 (3)	C60—H60C	0.9600
C37A—N1A	1.44 (3)	C61—H61A	0.9600
C37A—H37A	0.9700	C61—H61B	0.9600
C37A—H37B	0.9700	C61—H61C	0.9600
N1A—H1A	0.8600	C62—H62A	0.9600
N2A—C39A	1.378 (13)	C62—H62B	0.9600
N2A—H2A1	0.8600	C62—H62C	0.9600
C39A—C40A	1.619 (12)	C63—H63A	0.9600
C39A—H39A	0.9700	C63—H63B	0.9600
C39A—H39B	0.9700	C63—H63C	0.9600
C40A—C45A	1.354 (7)	C64—H64A	0.9600
C40A—C41A	1.369 (10)	C64—H64B	0.9600
C41A—C42A	1.348 (11)	C64—H64C	0.9600
C41A—H41A	0.9300	C65—H65A	0.9600
C42A—C43A	1.325 (12)	C65—H65B	0.9600
C42A—H42A	0.9300	C65—H65C	0.9600
C43A—C44A	1.391 (11)	C66—H66	0.9300
C43A—H43A	0.9300		
			
C3—O1—C36	118.0 (2)	N1B—C37B—H37C	111.9
C6—O3—C46	118.1 (2)	C36—C37B—H37C	111.9
C10—O5—C56	118.9 (2)	N1B—C37B—H37D	111.9
C13—O6—C57	118.3 (2)	C36—C37B—H37D	111.9
C17—O7—C58	118.7 (2)	H37C—C37B—H37D	109.6
C20—O8—C59	118.7 (3)	C38B—N1B—C37B	107.8 (19)
C24—O9—C60	119.6 (3)	C38B—N1B—H1B	126.1
C61—O10—C27	117.6 (3)	C37B—N1B—H1B	126.1
C62—O11—C31	119.3 (3)	C39B—N2B—C38B	115.8 (14)
C34—O12—C63	118.0 (3)	C39B—N2B—H2B	122.1
C66—N5—C65	122.0 (3)	C38B—N2B—H2B	122.1
C66—N5—C64	119.2 (3)	N2B—C39B—C40B	109.0 (11)
C65—N5—C64	118.8 (3)	N2B—C39B—H39C	109.9
C6—C1—C2	117.9 (2)	C40B—C39B—H39C	109.9
C6—C1—C35	122.0 (2)	N2B—C39B—H39D	109.9
C2—C1—C35	120.0 (2)	C40B—C39B—H39D	109.9
C1—C2—C3	121.7 (2)	H39C—C39B—H39D	108.3
C1—C2—H2A	119.2	C41B—C40B—C45B	120.0
C3—C2—H2A	119.2	C41B—C40B—C39B	92.7 (9)
O1—C3—C4	115.9 (2)	C45B—C40B—C39B	147.3 (9)
O1—C3—C2	123.9 (2)	C42B—C41B—C40B	120.0
C4—C3—C2	120.2 (2)	C42B—C41B—H41B	120.0
C5—C4—C3	117.9 (2)	C40B—C41B—H41B	120.0
C5—C4—C7	120.8 (2)	C43B—C42B—C41B	120.0
C3—C4—C7	121.3 (2)	C43B—C42B—H42B	120.0
C4—C5—C6	121.7 (2)	C41B—C42B—H42B	120.0
C4—C5—H5	119.2	C42B—C43B—C44B	120.0
C6—C5—H5	119.2	C42B—C43B—H43B	120.0
C1—C6—O3	116.4 (2)	C44B—C43B—H43B	120.0
C1—C6—C5	120.6 (2)	C43B—C44B—C45B	120.0
O3—C6—C5	122.9 (2)	C43B—C44B—H44B	120.0
C8—C7—C4	112.67 (19)	C45B—C44B—H44B	120.0
C8—C7—H7A	109.1	C44B—C45B—C40B	120.0
C4—C7—H7A	109.1	C44B—C45B—H45B	120.0
C8—C7—H7B	109.1	C40B—C45B—H45B	120.0
C4—C7—H7B	109.1	O3—C46—C47A	115.1 (6)
H7A—C7—H7B	107.8	O3—C46—C47B	100.0 (6)
C9—C8—C13	117.4 (2)	O3—C46—H46A	108.5
C9—C8—C7	121.6 (2)	C47A—C46—H46A	108.5
C13—C8—C7	121.0 (2)	O3—C46—H46B	108.5
C8—C9—C10	122.0 (2)	C47A—C46—H46B	108.5
C8—C9—H9	119.0	H46A—C46—H46B	107.5
C10—C9—H9	119.0	O3—C46—H46C	111.8
C11—C10—O5	115.9 (3)	C47B—C46—H46C	111.8
C11—C10—C9	120.2 (2)	O3—C46—H46D	111.8
O5—C10—C9	123.9 (2)	C47B—C46—H46D	111.8
C10—C11—C12	118.0 (3)	H46C—C46—H46D	109.5
C10—C11—C14	122.1 (3)	O4A—C48A—N3A	125 (2)
C12—C11—C14	119.8 (2)	O4A—C48A—N4A	127 (2)
C13—C12—C11	121.8 (2)	N3A—C48A—N4A	108.3 (15)
C13—C12—H12	119.1	N3A—C47A—C46	108.8 (11)
C11—C12—H12	119.1	N3A—C47A—H47A	109.9
C12—C13—O6	124.0 (2)	C46—C47A—H47A	109.9
C12—C13—C8	120.5 (3)	N3A—C47A—H47B	109.9
O6—C13—C8	115.5 (3)	C46—C47A—H47B	109.9
C11—C14—C15	111.3 (2)	H47A—C47A—H47B	108.3
C11—C14—H14A	109.4	C48A—N3A—C47A	113.5 (15)
C15—C14—H14A	109.4	C48A—N3A—H3A	123.2
C11—C14—H14B	109.4	C47A—N3A—H3A	123.2
C15—C14—H14B	109.4	C49A—N4A—C48A	120.3 (16)
H14A—C14—H14B	108.0	C49A—N4A—H4A	119.9
C16—C15—C20	117.6 (2)	C48A—N4A—H4A	119.9
C16—C15—C14	120.4 (2)	N4A—C49A—C50A	111.9 (8)
C20—C15—C14	121.9 (3)	N4A—C49A—H49A	109.2
C15—C16—C17	121.8 (3)	C50A—C49A—H49A	109.2
C15—C16—H16	119.1	N4A—C49A—H49B	109.2
C17—C16—H16	119.1	C50A—C49A—H49B	109.2
O7—C17—C18	115.3 (3)	H49A—C49A—H49B	107.9
O7—C17—C16	124.3 (3)	C51A—C50A—C55A	120.0
C18—C17—C16	120.4 (3)	C51A—C50A—C49A	123.1 (6)
C19—C18—C17	117.7 (3)	C55A—C50A—C49A	116.9 (6)
C19—C18—C21	121.2 (3)	C50A—C51A—C52A	120.0
C17—C18—C21	121.1 (3)	C50A—C51A—H51A	120.0
C18—C19—C20	121.9 (3)	C52A—C51A—H51A	120.0
C18—C19—H19	119.0	C51A—C52A—C53A	120.0
C20—C19—H19	119.0	C51A—C52A—H52A	120.0
O8—C20—C15	116.0 (3)	C53A—C52A—H52A	120.0
O8—C20—C19	123.5 (3)	C54A—C53A—C52A	120.0
C15—C20—C19	120.5 (3)	C54A—C53A—H53A	120.0
C22—C21—C18	113.0 (2)	C52A—C53A—H53A	120.0
C22—C21—H21A	109.0	C55A—C54A—C53A	120.0
C18—C21—H21A	109.0	C55A—C54A—H54A	120.0
C22—C21—H21B	109.0	C53A—C54A—H54A	120.0
C18—C21—H21B	109.0	C54A—C55A—C50A	120.0
H21A—C21—H21B	107.8	C54A—C55A—H55A	120.0
C27—C22—C23	117.7 (3)	C50A—C55A—H55A	120.0
C27—C22—C21	120.8 (3)	O4B—C48B—N4B	121 (3)
C23—C22—C21	121.5 (3)	O4B—C48B—N3B	118 (2)
C24—C23—C22	121.0 (3)	N4B—C48B—N3B	120.1 (18)
C24—C23—H23	119.5	N3B—C47B—C46	115.3 (12)
C22—C23—H23	119.5	N3B—C47B—H47C	108.4
C25—C24—O9	116.3 (3)	C46—C47B—H47C	108.4
C25—C24—C23	120.8 (3)	N3B—C47B—H47D	108.4
O9—C24—C23	122.9 (3)	C46—C47B—H47D	108.4
C24—C25—C26	118.2 (3)	H47C—C47B—H47D	107.5
C24—C25—C28	121.4 (3)	C48B—N3B—C47B	127.8 (16)
C26—C25—C28	120.4 (3)	C48B—N3B—H3B	116.1
C25—C26—C27	121.1 (3)	C47B—N3B—H3B	116.1
C25—C26—H26	119.4	C48B—N4B—C49B	123.2 (19)
C27—C26—H26	119.4	C48B—N4B—H4B	118.4
C22—C27—C26	121.1 (3)	C49B—N4B—H4B	118.4
C22—C27—O10	116.3 (3)	N4B—C49B—C50B	113.6 (10)
C26—C27—O10	122.7 (3)	N4B—C49B—H49C	108.8
C29—C28—C25	112.1 (2)	C50B—C49B—H49C	108.8
C29—C28—H28A	109.2	N4B—C49B—H49D	108.8
C25—C28—H28A	109.2	C50B—C49B—H49D	108.8
C29—C28—H28B	109.2	H49C—C49B—H49D	107.7
C25—C28—H28B	109.2	C51B—C50B—C55B	114.9 (10)
H28A—C28—H28B	107.9	C51B—C50B—C49B	125.5 (9)
C30—C29—C34	117.1 (3)	C55B—C50B—C49B	119.6 (10)
C30—C29—C28	121.8 (3)	C52B—C51B—C50B	126.9 (10)
C34—C29—C28	121.0 (3)	C52B—C51B—H51B	116.6
C29—C30—C31	121.8 (3)	C50B—C51B—H51B	116.6
C29—C30—H30	119.1	C53B—C52B—C51B	119.1 (11)
C31—C30—H30	119.1	C53B—C52B—H52B	120.4
C32—C31—C30	121.1 (3)	C51B—C52B—H52B	120.4
C32—C31—O11	115.5 (3)	C52B—C53B—C54B	119.7 (12)
C30—C31—O11	123.4 (3)	C52B—C53B—H53B	120.2
C31—C32—C33	117.4 (3)	C54B—C53B—H53B	120.2
C31—C32—C35	122.1 (3)	C53B—C54B—C55B	120.1 (12)
C33—C32—C35	120.4 (3)	C53B—C54B—H54B	119.9
C34—C33—C32	121.4 (3)	C55B—C54B—H54B	119.9
C34—C33—H33	119.3	C50B—C55B—C54B	119.0 (10)
C32—C33—H33	119.3	C50B—C55B—H55B	120.5
C33—C34—O12	123.3 (3)	C54B—C55B—H55B	120.5
C33—C34—C29	121.1 (3)	O5—C56—H56A	109.5
O12—C34—C29	115.6 (3)	O5—C56—H56B	109.5
C32—C35—C1	111.5 (2)	H56A—C56—H56B	109.5
C32—C35—H35A	109.3	O5—C56—H56C	109.5
C1—C35—H35A	109.3	H56A—C56—H56C	109.5
C32—C35—H35B	109.3	H56B—C56—H56C	109.5
C1—C35—H35B	109.3	O6—C57—H57A	109.5
H35A—C35—H35B	108.0	O6—C57—H57B	109.5
C37A—C36—O1	110.6 (8)	H57A—C57—H57B	109.5
O1—C36—C37B	102.9 (9)	O6—C57—H57C	109.5
C37A—C36—H36A	109.5	H57A—C57—H57C	109.5
O1—C36—H36A	109.5	H57B—C57—H57C	109.5
C37A—C36—H36B	109.5	O7—C58—H58A	109.5
O1—C36—H36B	109.5	O7—C58—H58B	109.5
H36A—C36—H36B	108.1	H58A—C58—H58B	109.5
O1—C36—H36C	111.2	O7—C58—H58C	109.5
C37B—C36—H36C	111.2	H58A—C58—H58C	109.5
O1—C36—H36D	111.2	H58B—C58—H58C	109.5
C37B—C36—H36D	111.2	O8—C59—H59A	109.5
H36C—C36—H36D	109.1	O8—C59—H59B	109.5
O2A—C38A—N2A	114.7 (19)	H59A—C59—H59B	109.5
O2A—C38A—N1A	117.8 (18)	O8—C59—H59C	109.5
N2A—C38A—N1A	124.9 (16)	H59A—C59—H59C	109.5
C36—C37A—N1A	118.9 (14)	H59B—C59—H59C	109.5
C36—C37A—H37A	107.6	O9—C60—H60A	109.5
N1A—C37A—H37A	107.6	O9—C60—H60B	109.5
C36—C37A—H37B	107.6	H60A—C60—H60B	109.5
N1A—C37A—H37B	107.6	O9—C60—H60C	109.5
H37A—C37A—H37B	107.0	H60A—C60—H60C	109.5
C38A—N1A—C37A	127.2 (18)	H60B—C60—H60C	109.5
C38A—N1A—H1A	116.4	O10—C61—H61A	109.5
C37A—N1A—H1A	116.4	O10—C61—H61B	109.5
C38A—N2A—C39A	127.2 (14)	H61A—C61—H61B	109.5
C38A—N2A—H2A1	116.4	O10—C61—H61C	109.5
C39A—N2A—H2A1	116.4	H61A—C61—H61C	109.5
N2A—C39A—C40A	110.5 (8)	H61B—C61—H61C	109.5
N2A—C39A—H39A	109.6	O11—C62—H62A	109.5
C40A—C39A—H39A	109.6	O11—C62—H62B	109.5
N2A—C39A—H39B	109.6	H62A—C62—H62B	109.5
C40A—C39A—H39B	109.6	O11—C62—H62C	109.5
H39A—C39A—H39B	108.1	H62A—C62—H62C	109.5
C45A—C40A—C41A	118.3 (7)	H62B—C62—H62C	109.5
C45A—C40A—C39A	96.9 (9)	O12—C63—H63A	109.5
C41A—C40A—C39A	143.4 (9)	O12—C63—H63B	109.5
C42A—C41A—C40A	122.4 (8)	H63A—C63—H63B	109.5
C42A—C41A—H41A	118.8	O12—C63—H63C	109.5
C40A—C41A—H41A	118.8	H63A—C63—H63C	109.5
C43A—C42A—C41A	118.9 (9)	H63B—C63—H63C	109.5
C43A—C42A—H42A	120.6	N5—C64—H64A	109.5
C41A—C42A—H42A	120.6	N5—C64—H64B	109.5
C42A—C43A—C44A	121.6 (9)	H64A—C64—H64B	109.5
C42A—C43A—H43A	119.2	N5—C64—H64C	109.5
C44A—C43A—H43A	119.2	H64A—C64—H64C	109.5
C45A—C44A—C43A	118.0 (9)	H64B—C64—H64C	109.5
C45A—C44A—H44A	121.0	N5—C65—H65A	109.5
C43A—C44A—H44A	121.0	N5—C65—H65B	109.5
C40A—C45A—C44A	120.5 (8)	H65A—C65—H65B	109.5
C40A—C45A—H45A	119.8	N5—C65—H65C	109.5
C44A—C45A—H45A	119.8	H65A—C65—H65C	109.5
O2B—C38B—N1B	130 (3)	H65B—C65—H65C	109.5
O2B—C38B—N2B	134 (2)	O13—C66—N5	126.6 (3)
N1B—C38B—N2B	93.7 (13)	O13—C66—H66	116.7
N1B—C37B—C36	99.4 (15)	N5—C66—H66	116.7
			
C6—C1—C2—C3	0.9 (4)	C28—C29—C30—C31	−175.7 (3)
C35—C1—C2—C3	−178.8 (2)	C29—C30—C31—C32	−0.6 (4)
C36—O1—C3—C4	−163.3 (2)	C29—C30—C31—O11	178.0 (3)
C36—O1—C3—C2	15.7 (3)	C62—O11—C31—C32	176.1 (4)
C1—C2—C3—O1	−179.3 (2)	C62—O11—C31—C30	−2.6 (5)
C1—C2—C3—C4	−0.4 (4)	C30—C31—C32—C33	−1.2 (4)
O1—C3—C4—C5	178.6 (2)	O11—C31—C32—C33	−179.9 (2)
C2—C3—C4—C5	−0.4 (3)	C30—C31—C32—C35	176.6 (3)
O1—C3—C4—C7	−1.2 (3)	O11—C31—C32—C35	−2.2 (4)
C2—C3—C4—C7	179.8 (2)	C31—C32—C33—C34	1.6 (4)
C3—C4—C5—C6	0.7 (4)	C35—C32—C33—C34	−176.2 (3)
C7—C4—C5—C6	−179.5 (2)	C32—C33—C34—O12	−179.7 (3)
C2—C1—C6—O3	177.2 (2)	C32—C33—C34—C29	−0.2 (4)
C35—C1—C6—O3	−3.1 (3)	C63—O12—C34—C33	−8.4 (5)
C2—C1—C6—C5	−0.6 (4)	C63—O12—C34—C29	172.1 (3)
C35—C1—C6—C5	179.1 (2)	C30—C29—C34—C33	−1.5 (4)
C46—O3—C6—C1	167.2 (2)	C28—C29—C34—C33	176.1 (3)
C46—O3—C6—C5	−15.1 (4)	C30—C29—C34—O12	177.9 (2)
C4—C5—C6—C1	−0.2 (4)	C28—C29—C34—O12	−4.4 (4)
C4—C5—C6—O3	−177.9 (2)	C31—C32—C35—C1	−108.0 (3)
C5—C4—C7—C8	100.6 (3)	C33—C32—C35—C1	69.7 (3)
C3—C4—C7—C8	−79.7 (3)	C6—C1—C35—C32	−96.1 (3)
C4—C7—C8—C9	107.8 (3)	C2—C1—C35—C32	83.6 (3)
C4—C7—C8—C13	−70.8 (3)	C3—O1—C36—C37A	174.3 (8)
C13—C8—C9—C10	1.4 (3)	C3—O1—C36—C37B	171.0 (9)
C7—C8—C9—C10	−177.2 (2)	O1—C36—C37A—N1A	65.5 (19)
C56—O5—C10—C11	−173.5 (2)	O2A—C38A—N1A—C37A	31 (3)
C56—O5—C10—C9	8.8 (4)	N2A—C38A—N1A—C37A	−168.7 (14)
C8—C9—C10—C11	0.6 (4)	C36—C37A—N1A—C38A	129.7 (18)
C8—C9—C10—O5	178.1 (2)	O2A—C38A—N2A—C39A	−13 (2)
O5—C10—C11—C12	−179.6 (2)	N1A—C38A—N2A—C39A	−174.8 (14)
C9—C10—C11—C12	−1.9 (4)	C38A—N2A—C39A—C40A	−169.2 (16)
O5—C10—C11—C14	−1.7 (3)	N2A—C39A—C40A—C45A	−84.5 (9)
C9—C10—C11—C14	176.1 (2)	N2A—C39A—C40A—C41A	111.0 (15)
C10—C11—C12—C13	1.3 (3)	C45A—C40A—C41A—C42A	−3.8 (13)
C14—C11—C12—C13	−176.8 (2)	C39A—C40A—C41A—C42A	158.7 (16)
C11—C12—C13—O6	−178.1 (2)	C40A—C41A—C42A—C43A	0.4 (14)
C11—C12—C13—C8	0.7 (4)	C41A—C42A—C43A—C44A	0.5 (16)
C57—O6—C13—C12	−9.3 (4)	C42A—C43A—C44A—C45A	2.1 (15)
C57—O6—C13—C8	171.9 (2)	C41A—C40A—C45A—C44A	6.4 (13)
C9—C8—C13—C12	−2.1 (3)	C39A—C40A—C45A—C44A	−163.2 (10)
C7—C8—C13—C12	176.6 (2)	C43A—C44A—C45A—C40A	−5.6 (13)
C9—C8—C13—O6	176.8 (2)	O1—C36—C37B—N1B	83.8 (16)
C7—C8—C13—O6	−4.5 (3)	O2B—C38B—N1B—C37B	−10 (4)
C10—C11—C14—C15	−105.6 (3)	N2B—C38B—N1B—C37B	−176.1 (16)
C12—C11—C14—C15	72.4 (3)	C36—C37B—N1B—C38B	150.1 (17)
C11—C14—C15—C16	76.2 (3)	O2B—C38B—N2B—C39B	−7 (3)
C11—C14—C15—C20	−99.3 (3)	N1B—C38B—N2B—C39B	158.9 (13)
C20—C15—C16—C17	0.1 (4)	C38B—N2B—C39B—C40B	131.9 (15)
C14—C15—C16—C17	−175.5 (2)	N2B—C39B—C40B—C41B	117.4 (8)
C58—O7—C17—C18	178.1 (3)	N2B—C39B—C40B—C45B	−60 (2)
C58—O7—C17—C16	−3.0 (4)	C45B—C40B—C41B—C42B	0.0
C15—C16—C17—O7	−180.0 (3)	C39B—C40B—C41B—C42B	−178.7 (11)
C15—C16—C17—C18	−1.2 (4)	C40B—C41B—C42B—C43B	0.0
O7—C17—C18—C19	179.6 (2)	C41B—C42B—C43B—C44B	0.0
C16—C17—C18—C19	0.7 (4)	C42B—C43B—C44B—C45B	0.0
O7—C17—C18—C21	0.0 (4)	C43B—C44B—C45B—C40B	0.0
C16—C17—C18—C21	−179.0 (3)	C41B—C40B—C45B—C44B	0.0
C17—C18—C19—C20	0.8 (4)	C39B—C40B—C45B—C44B	178 (2)
C21—C18—C19—C20	−179.6 (3)	C6—O3—C46—C47A	178.3 (7)
C59—O8—C20—C15	158.8 (3)	C6—O3—C46—C47B	−178.0 (8)
C59—O8—C20—C19	−23.1 (5)	O3—C46—C47A—N3A	−68.7 (10)
C16—C15—C20—O8	179.5 (3)	O4A—C48A—N3A—C47A	9 (3)
C14—C15—C20—O8	−4.9 (4)	N4A—C48A—N3A—C47A	−170.5 (15)
C16—C15—C20—C19	1.4 (4)	C46—C47A—N3A—C48A	−155.8 (13)
C14—C15—C20—C19	176.9 (3)	O4A—C48A—N4A—C49A	−9 (3)
C18—C19—C20—O8	−179.8 (3)	N3A—C48A—N4A—C49A	169.7 (11)
C18—C19—C20—C15	−1.8 (5)	C48A—N4A—C49A—C50A	154.7 (16)
C19—C18—C21—C22	94.6 (3)	N4A—C49A—C50A—C51A	−60.2 (10)
C17—C18—C21—C22	−85.8 (4)	N4A—C49A—C50A—C55A	119.9 (9)
C18—C21—C22—C27	−78.9 (3)	C55A—C50A—C51A—C52A	0.0
C18—C21—C22—C23	99.1 (3)	C49A—C50A—C51A—C52A	−179.9 (8)
C27—C22—C23—C24	1.6 (4)	C50A—C51A—C52A—C53A	0.0
C21—C22—C23—C24	−176.5 (2)	C51A—C52A—C53A—C54A	0.0
C60—O9—C24—C25	−169.5 (3)	C52A—C53A—C54A—C55A	0.0
C60—O9—C24—C23	12.6 (5)	C53A—C54A—C55A—C50A	0.0
C22—C23—C24—C25	−0.1 (4)	C51A—C50A—C55A—C54A	0.0
C22—C23—C24—O9	177.6 (2)	C49A—C50A—C55A—C54A	179.9 (8)
O9—C24—C25—C26	−179.4 (2)	O3—C46—C47B—N3B	−80.3 (11)
C23—C24—C25—C26	−1.5 (4)	O4B—C48B—N3B—C47B	−12 (3)
O9—C24—C25—C28	0.0 (4)	N4B—C48B—N3B—C47B	162 (2)
C23—C24—C25—C28	177.9 (2)	C46—C47B—N3B—C48B	−127.3 (17)
C24—C25—C26—C27	1.7 (4)	O4B—C48B—N4B—C49B	2 (4)
C28—C25—C26—C27	−177.7 (2)	N3B—C48B—N4B—C49B	−172.7 (13)
C23—C22—C27—C26	−1.4 (4)	C48B—N4B—C49B—C50B	−155 (2)
C21—C22—C27—C26	176.7 (2)	N4B—C49B—C50B—C51B	−121.5 (13)
C23—C22—C27—O10	177.7 (2)	N4B—C49B—C50B—C55B	61.1 (15)
C21—C22—C27—O10	−4.2 (4)	C55B—C50B—C51B—C52B	−1.3 (16)
C25—C26—C27—C22	−0.2 (4)	C49B—C50B—C51B—C52B	−178.8 (11)
C25—C26—C27—O10	−179.3 (2)	C50B—C51B—C52B—C53B	1.2 (17)
C61—O10—C27—C22	153.1 (3)	C51B—C52B—C53B—C54B	−3.7 (17)
C61—O10—C27—C26	−27.8 (4)	C52B—C53B—C54B—C55B	6.4 (17)
C24—C25—C28—C29	−89.6 (3)	C51B—C50B—C55B—C54B	3.8 (15)
C26—C25—C28—C29	89.8 (3)	C49B—C50B—C55B—C54B	−178.5 (9)
C25—C28—C29—C30	98.3 (3)	C53B—C54B—C55B—C50B	−6.5 (16)
C25—C28—C29—C34	−79.2 (4)	C65—N5—C66—O13	178.6 (3)
C34—C29—C30—C31	2.0 (4)	C64—N5—C66—O13	−2.5 (5)
